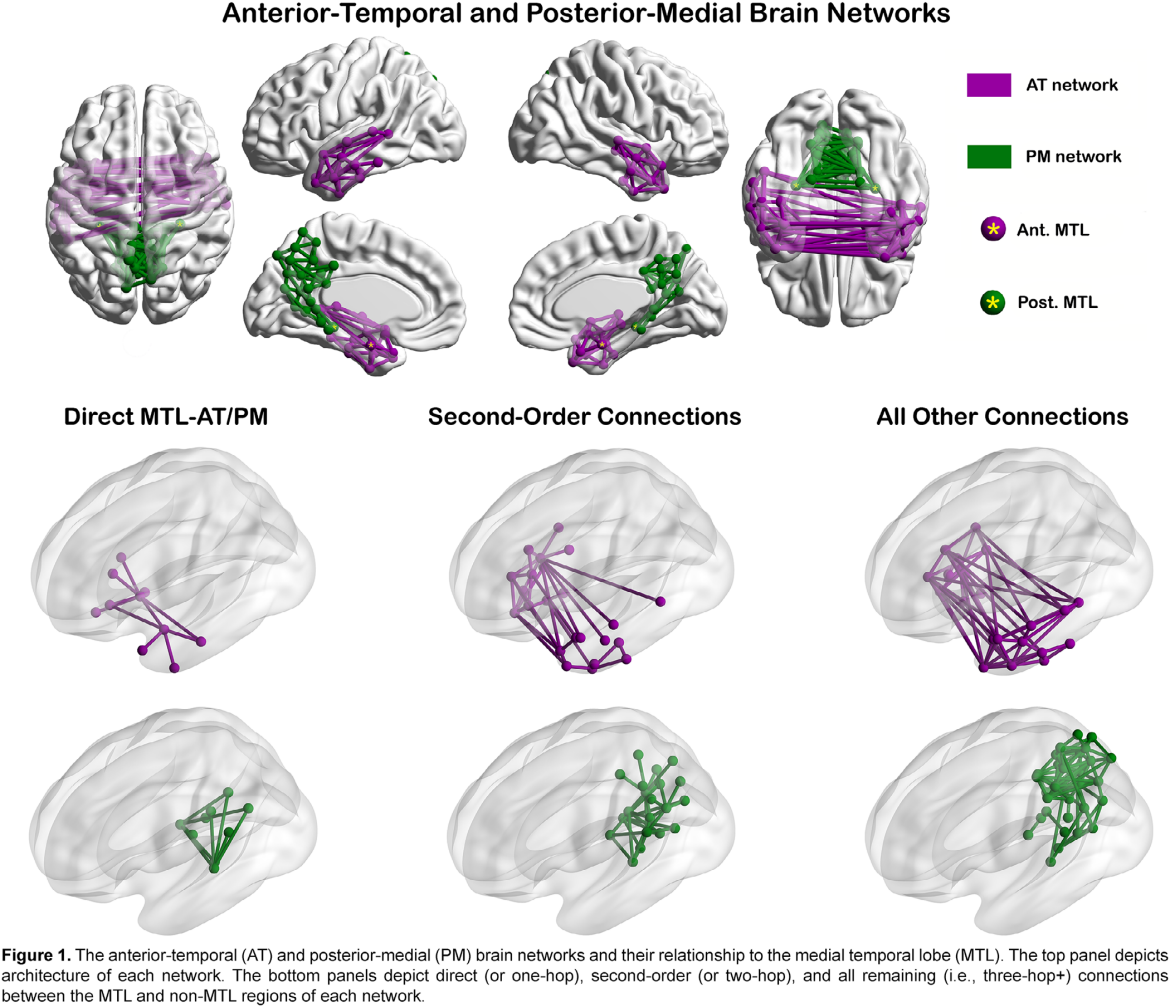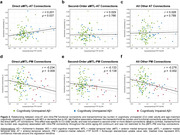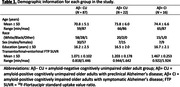# MTL networks are differentially affected by tau pathology

**DOI:** 10.1002/alz.092666

**Published:** 2025-01-09

**Authors:** Stanislau Hrybouski, Sandhitsu R. Das, Long Xie, Laura E.M. Wisse, Melissa Kelley, Jacqueline Lane, Monica Sherin, Michael DiCalogero, Ilya M. Nasrallah, John A. Detre, Paul A. Yushkevich, David A Wolk

**Affiliations:** ^1^ University of Pennsylvania, Philadelphia, PA USA; ^2^ Penn Image Computing and Science Laboratory (PICSL), University of Pennsylvania, Philadelphia, PA USA; ^3^ Penn Alzheimer’s Disease Research Center, University of Pennsylvania, Philadelphia, PA USA; ^4^ Lund University, Lund Sweden

## Abstract

**Background:**

The extent to which pathological processes in aging and Alzheimer’s disease (AD) relate to functional alterations in the medial temporal lobe (MTL)‐dependent brain networks is poorly understood. Here, we examined the relationship between tau accumulation in the (trans)entorhinal cortex and functional connectivity (FC) in two MTL‐affiliated brain networks — the Anterior‐Temporal (AT) and Posterior‐Medial (PM) — in normal agers, individuals with preclinical AD, and patients with symptomatic AD.

**Method:**

In this cross‐sectional study, we analyzed data from 125 individuals from the Penn ADRC (Table 1). All participants underwent structural and functional MRI on a Siemens 3 T Prisma system, as well as ^18^F‐Florbetaben and ^18^F‐Flortaucipir (FTP) PET imaging. We used the AT/PM network architecture from our previous work (Figure 1; PMID37767219) and analyzed the effects of (trans)entorhinal tau accumulation on the AT/PM FC as a function of distance to the MTL (Figure 1). Guided by our previous findings of an inverted U‐shaped FC pattern in the AT network over the course of the disease, we performed separate analyses of the AT FC in cognitively normal and symptomatic individuals.

**Results:**

FC between the anterior MTL and its direct neighbors in the AT network was positively correlated with tau accumulation in the MTL (r = 0.201, p = 0.031; Figure 2a). Associations with tau were not present in other MTL‐AT connections (Figures 2b‐c) or in individuals with symptomatic AD. In contrast, the PM FC was broadly affected by tau pathology with both direct MTL‐PM and more distant connections showing a negative relationship to (trans)entorhinal tau (Figure 2d‐e). Excluding amyloid‐negative controls, the relationship between MTL tau and AT/PM FC remained statistically significant in the PM [one‐hop connections: r = ‐0.323, p = 0.0479; distant connections: r = ‐0.324, p = 0.047] but not the AT network.

**Conclusions:**

Together, the current results implicate functional abnormalities in the AT network during the preclinical stage, while those in the PM network are closely related to disease severity. This dissociation likely represents distinct pathophysiology in AD and has potential implications for FC‐based metrics as a surrogate measure for assessing functional response to disease‐modifying immunotherapies.